# Spatial Deep Learning Approach to Older Driver Classification

**DOI:** 10.1109/access.2024.3516572

**Published:** 2024-12-12

**Authors:** CHARLES BOATENG, SEYEDEH GOL ARA GHOREISHI, KWANGSOO YANG, MUHAMMAD TANVEER JAN, RUTH TAPPEN, JINWOO JANG, DAVID NEWMAN, SONIA MOSHFEGHI, KELLY JACKSON, RHIAN RESNICK, BORKO FURHT, MONICA ROSSELLI, JOSHUA CONNIFF

**Affiliations:** 1 Florida Atlantic University, Boca Raton, USA; 2 Florida Atlantic University, Boca Raton, USA; 3 Florida Atlantic University, Boca Raton, USA; 4 Florida Atlantic University, Boca Raton, USA; 5 Florida Atlantic University, Boca Raton, USA; 6 Florida Atlantic University, Boca Raton, USA; 7 Florida Atlantic University, Boca Raton, USA; 8 Florida Atlantic University, Boca Raton, USA; 9 Florida Atlantic University, Boca Raton, USA; 10 Florida Atlantic University, Boca Raton, USA; 11 Florida Atlantic University, Boca Raton, USA; 12 Florida Atlantic University, Boca Raton, USA; 13 Florida Atlantic University, Boca Raton, USA

**Keywords:** Spatial Deep Learning, Older Driver Classification, Trajectory Data Mining

## Abstract

Given telemetry datasets (e.g., GPS location, speed, direction, distance.), the Older Driver Classification (ODC) problem identifies two groups of drivers: normal and abnormal. The ODC problem is essential in many societal applications, including road safety, insurance risk assessment, and targeted interventions for elderly drivers with dementia or Mild Cognitive Impairment (MCI). The problem is challenging because of the volume and heterogeneity of temporally-detailed vehicle datasets. This paper proposes a novel spatial deep-learning approach that leverages Grid-Index based data augmentation to enhance the detection of abnormal driving behaviors. Through extensive experiments and a real-world case study, the proposed approach consistently identifies abnormal drivers with high accuracy. The findings demonstrate the potential of grid-based methods to improve telematics-based driving behavior analysis significantly. This approach offers valuable implications for enhancing road safety measures, optimizing insurance risk assessments, and developing targeted interventions for at-risk drivers.

## I. INTRODUCTION

Given telemetry datasets (e.g., GPS location, speed, direction), the Older Driver Classification (ODC) problem identifies two groups of drivers: normal and abnormal. The ODC problem plays a significant role in road safety improvement and targeted interventions for elderly drivers with dementia or Mild Cognitive Impairment (MCI). This problem is challenging due to the volume and heterogeneity of data collected from vehicles and the complexity of spatial-temporal patterns in driving behavior. Traditional classification methods often rely solely on basic telematics features such as speed, direction, and distance, which lack the granularity to capture subtle driving anomalies that may indicate cognitive decline, such as delayed braking or inconsistent lane changes. To address this, we introduce a novel method that combines traditional features with advanced spatial-temporal analysis to improve classification accuracy.

Our study leverages grid-indexed shape analysis to enhance the accuracy of vehicle trajectory classification. The core idea is to segment trajectories with a time window and grid cells and utilize temporally detailed spatial grid indexes. This approach allows convolution filters to recognize complex spatiotemporal patterns.

**FIGURE 1:**
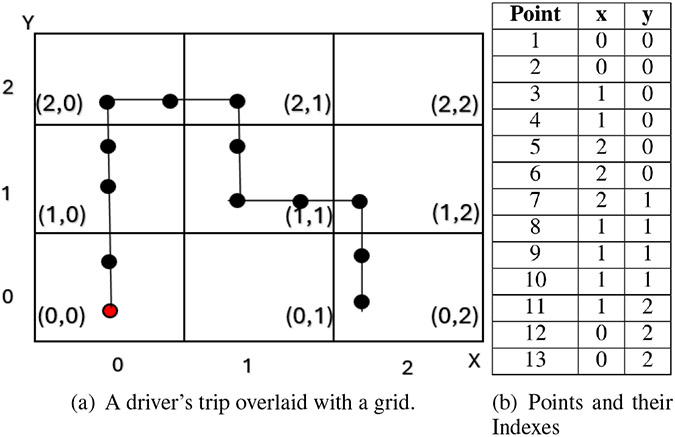
Example of a driver’s trip

We first decompose the trajectory into segments based on a fixed time window. Assume that the trajectory takes n units of time. Let the size of the time window be w. Then, the number of segments is n−w+1. Figure 1(a) shows an example of a segment of the trajectory overlaid with grids. The red dot indicates the starting point of the trip. In this example, we assume that the time window size is 13 units. Figure 1(b) shows an example of points and their indexes for the segment shown in Figure 1(a). The grid id (x,y) represents the coordinates, where x is the index along the longitude axis and y is the index along the latitude axis.

Our method captures complex spatiotemporal driving patterns with these grid-indexed segments, enabling more accurate classification of normal and abnormal driving behaviors. By analyzing these grid indexes, we can detect driving behaviors that are not apparent through primary telematics data(e.g., GPS location, acceleration, direction, distance.) alone. Our proposed approach combines the strengths of traditional telematics features with advanced grid-based analysis to improve overall classification performance.

### A. APPLICATION DOMAIN

The Older Driver Classification (ODC) problem addresses the critical need to enhance road safety for elderly drivers, particularly those with Mild Cognitive Impairment (MCI) or dementia. The ODC problem leverages telematics technology to monitor and analyze driving patterns, crucial for identifying and mitigating abnormal driving behaviors that pose risks to drivers and others on the road.

**FIGURE 2:**
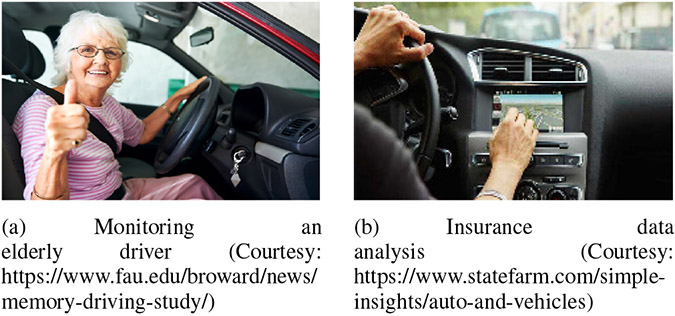
Applications of the ODC problem

Telematics data provides comprehensive insights into driving behaviors by capturing speed, direction, and location metrics. In the context of ODC, this data is instrumental in detecting cognitive decline in elderly drivers, indicated by behaviors like confusion at intersections, erratic speed changes, and missed turns. Early identification of these patterns allows family members and healthcare providers to intervene, ensuring the safety of elderly drivers and maintaining their independence and mobility [1]. Figure 2(a) illustrates the monitoring process of an elderly driver using telematics.

In fleet management, addressing the ODC problem involves optimizing driver performance by identifying and mitigating inefficient driving behaviors. Telematics data helps fleet operators optimize routing, minimize fuel consumption, and enforce safety protocols, enhancing overall efficiency and safety [2].

The insurance industry benefits from addressing the ODC problem through more accurate risk assessments and premium calculations. Telematics data enables personalized, usage-based insurance (UBI) policies, rewarding safe driving habits and detecting fraudulent claims, which protects the integrity of the insurance system. Figure 2(b) demonstrates the analysis of insurance data through telematics.

Transportation safety authorities use insights from the ODC problem to develop targeted interventions to reduce accident rates. By identifying and mitigating risky behaviors like aggressive driving, rapid lane changes, and excessive speeding, authorities can implement public awareness campaigns and enhanced enforcement measures to improve public safety [3].

Urban planning and infrastructure development also benefit from solving the ODC problem. Analyzing traffic flow and congestion patterns using telematics data helps urban planners design more efficient transportation networks, optimize traffic signals, plan new roadways, and enhance public transit systems to improve the commuting experience for urban dwellers [4], [5].

In research and development, addressing the ODC problem supports the advancement of autonomous driving technologies. By providing real-world data on vehicle dynamics and driver behavior, telematics enables the development of algorithms that improve the safety and reliability of self-driving cars [6].

In summary, addressing the Older Driver Classification (ODC) problem has far-reaching implications across multiple domains. The comprehensive analysis of driving behaviors using telematics data is indispensable for improving road safety, optimizing fleet management, refining insurance risk assessments, and advancing transportation technologies. The interdisciplinary impact of solving the ODC problem underscores its transformative potential in shaping the future of mobility.

### B. PROBLEM FORMULATION

Our study aims to develop a predictive model based on a spatial deep-learning framework for detecting abnormal driving behavior. The problem is formulated as follows:

#### Input:

- a set of vehicle drivers with temporally-detailed telematics data (e.g., lon, lat, speed, direction, distance),
- a binary label for normal and abnormal drivers,
- the size of the time window w, and
- the size of the grid cell c

#### Output:

Trajectory Classification Model

#### Objective:

- Maximize the predictive performance to classify driving behavior.

#### Constraints:

- The model must generalize well to unseen data, ensuring robust performance.

### C. OUR CONTRIBUTION

In this paper, we introduce a novel spatial deep learning approach to the Older Driver Classification (ODC) problem using telematics data. Our approach leverages grid indexes and data augmentation to enhance the detection of abnormal driving behaviors. Specifically, our contributions are as follows:

- We introduce the ODC problem, classifying older drivers into normal and abnormal categories using telematics data.
- We propose the grid indexes and data augmentation to effectively analyze the temporally detailed telematics data.
- We collect and process the real-world trajectory data from 200 vehicles for three years.
- We experimentally validate our approach using real-world telematics datasets, demonstrating significant improvements over traditional anomaly detection methods.

### D. RELATED WORK

The study of driving behavior has evolved significantly, from primary telemetry analyses to complex models utilizing advanced machine learning and deep learning techniques.

Initial research primarily used traditional methods like speed and braking force analysis and threshold-based detection systems. These systems monitored basic parameters to identify deviations from established norms [7]-[9]. Despite their utility, these early models needed more flexibility and scope.

Researchers then shifted toward statistical methods, such as Gaussian Mixture Models (GMMs) and Principal Component Analysis (PCA). These methods used statistical inference to detect subtle patterns in driving data but relied heavily on manually extracted features, which limited their effectiveness [10]-[12].

The adoption of machine learning algorithms marked a significant advancement. Techniques like Support Vector Machines (SVMs) and Random Forests (RFs) classified driving styles and detected anomalies using broader features. However, these methods required deep expertise to select relevant features and fine-tune the models [13]-[15].

With the advent of big data and neural networks, Deep Neural Networks (DNNs) and Convolutional Neural Networks (CNNs) emerged as practical tools. These models autonomously learned complex, hierarchical features without manual feature engineering, directly extracting discriminative features from high-dimensional raw data, such as images from traffic cameras and sensors [16]-[19].

Recognizing the importance of spatial analysis in trajectory data, researchers employed techniques in spatial data mining (Spatial DM) to uncover complex patterns in movement data, providing deeper insights into spatial behaviors [20], [21]. Although effective in analyzing large datasets, these methods sometimes lacked the temporal depth to fully understand dynamic behaviors [22], [23].

Our work builds upon these foundations by integrating spatial-temporal pattern recognition with deep learning capabilities [24], [25]. We introduce Grid-Index Resolution (GIR), an approach inspired by advances in spatial tessellation and grid-based modeling in geographical information systems (GIS). By segmenting trajectories into grid cells, we capture the geometric properties of driving routes.

This research distinguishes itself by employing convolution filters to analyze these grid-indexed shapes—a novel application of these networks, typically used in image and video recognition. Our model effectively learns from the spatiotemporal patterns within these detailed grid cells, surpassing traditional and advanced machine-learning techniques.

**FIGURE 3:**
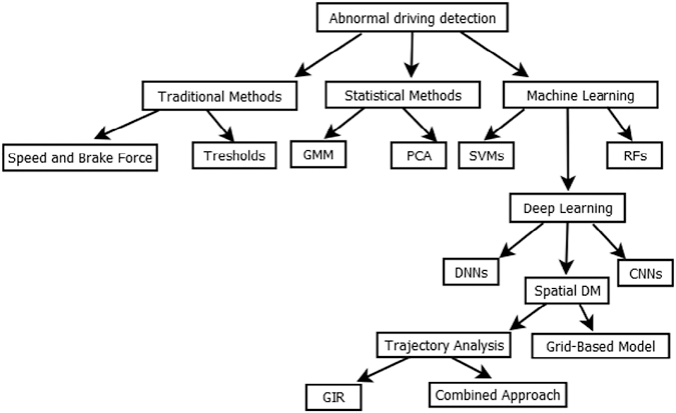
Approaches to the ODC problem.

Figure 3 illustrates the various approaches to the ODC problem, highlighting the evolution from traditional methods to advanced deep learning techniques.

### E. SCOPE AND OUTLINE

This paper organizes the remaining sections: Section II describes telematic data, grid indexes, and data augmentation. Section III introduces the proposed approach based on grid indexes and neural network models. Section IV presents the experimental observations and results. Finally, Section V concludes the paper.

## II. GRID-INDEX APPROACH

The Grid-Index Approach provides a systematic method for analyzing driving behavior using spatial and temporal dimensions. This approach transforms raw GPS data into a structured format for advanced pattern recognition using deep learning models. This section outlines the processes involved, from data collection to feature augmentation.

### A. DATA COLLECTION AND PREPARATION

We collect telematics data through precise GPS tracking, capturing every second of a vehicle’s journey. We calculate basic metrics such as distance, speed, and azimuth (or bearing) from GPS coordinates to extract meaningful features.

#### 1) Distance

The distance between two GPS points is calculated using the Haversine formula, which accounts for the Earth’s curvature. The formula is given by:

(1) $$d=2r\;\arcsin\left(\sqrt{\sin^{2}\left(\frac{\Delta \phi }{2}\right)+\cos(\phi_{1})\;\cos(\phi_{2})\;\sin^{2}\left(\frac{\Delta \lambda }{2}\right)}\right),$$

where r is the Earth’s radius, Δϕ is the difference in latitude, and Δλ is the difference in longitude between the two points $\left(\phi_{1},\lambda_{1}\right)$ and $\left(\phi_{2},\lambda_{2}\right)$.

#### 2) Speed

Given three GPS points, p1, p2, and p3, we can calculate the speed on p2 using the following equation.

(2) $$v(p2)=\frac{\Delta d_{p1p3}}{\Delta t_{p1p3}},$$

where $\Delta d_{p1p3}$ is the distance between p1 and p3 and $\Delta t_{p1p3}$ is the time interval between p1 and p3.

We also gathered speed over ground (SOG) values from the AutoPi device installed in the vehicles. We can enhance the learning model’s ability to understand speed patterns by utilizing both speed and SOG.

#### 3) Direction

We first compute the azimuth (or bearing) between two GPS points. However, the azimuth value for North is either 0 or 360 degrees, indicating a discontinuity. Our approach maps the azimuth value onto the unit circle and converts it to coordinates on the circle.

**FIGURE 4:**
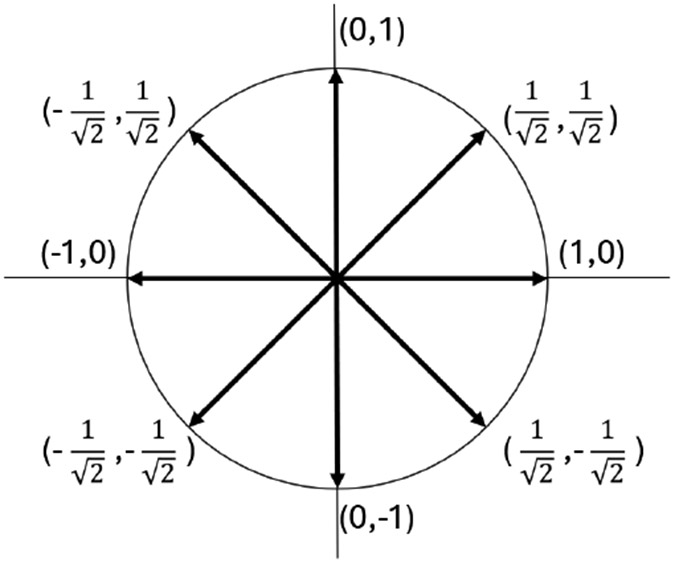
Representation of direction (azimuth) using cardinal and intercardinal directions.

Figure 4 shows an example of this transformation for cardinal and intercardinal directions. Table 1 shows an example of azimuth_x and azimuth_y values corresponding to the directions illustrated in Figure 1.

### B. SEGMENTATION AND AUGMENTATION

Our proposed approach involves three main steps: (1) segmenting a trajectory using a time window, (2) mapping each segment to the grid index, and (3) applying data augmentation through rotation to enhance model robustness.

First, we decompose the trajectory into segments based on the time window. Assume that the trajectory takes n units of time. Let the size of the time window be w. We slide the time window across the sequence of GPS points to create segments. If the sliding step equals one unit of time, then the number of segments is n−w+1.

Next, we map each segment to the grid index. We shift the starting point to the origin of the coordinate system and map each GPS point onto the grid index (see Figure 1). This shift-to-origin process ensures translation invariance.

**Lemma 1.** *The shift-to-origin process ensures translation invariance*.

*Proof*. The shift-to-origin process translates the input segment (or trajectory) so that the starting point becomes the origin of the coordinate system. Let S(x,y) be the input segment, and $S(x+\delta t_{x},y+\delta t_{y})$ be the translated segment. Then, both segments produce the same outcome after the shift-to-origin process. Thus, the proof is complete.

**FIGURE 5:**
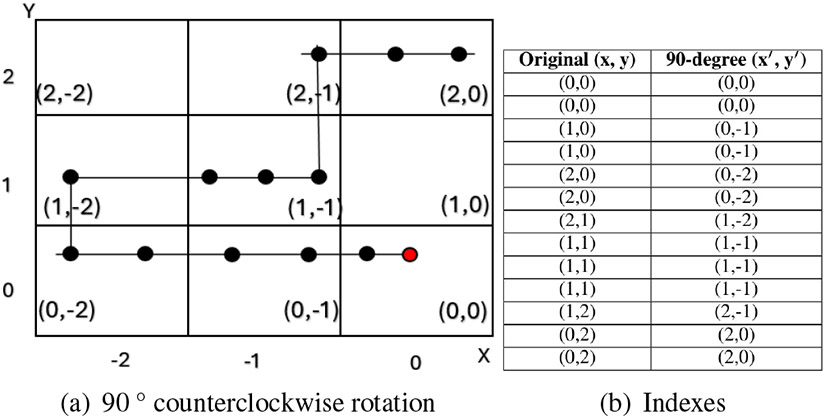
90 ° counterclockwise rotational transformation

**FIGURE 6:**
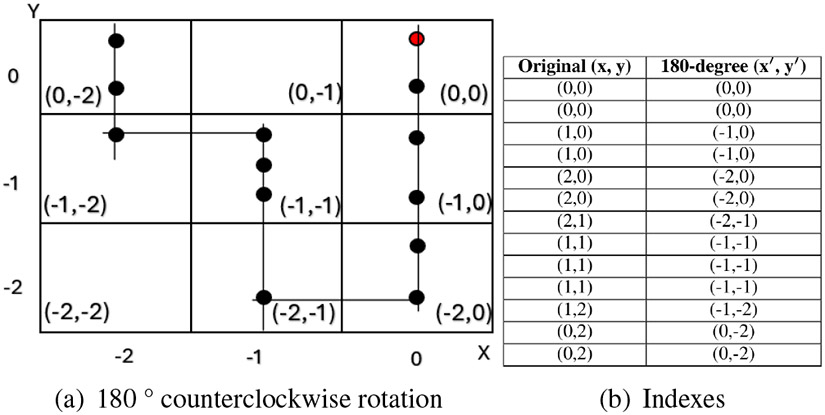
180 ° counterclockwise rotational transformation

Lastly, we perform data augmentation through rotation to increase the model’s capability to generalize across diverse driving conditions. This involves creating multiple rotated versions of each segment, which simulate different driving directions and orientations, allowing the model to recognize patterns regardless of the vehicle’s orientation. Specifically, we apply rotations of 90°, 180°, and 270°, as illustrated in Figure 5, 6, and 7. This rotation-based augmentation expands the dataset and ensures that the model captures a variety of spatial orientations, which is essential for detecting abnormal driving patterns across different directional movements.

**Lemma 2.** *The rotation process ensures rotation invariance*.

*Proof*. The rotation process augments the training data by including various rotated versions of the input segment. In our approach, we represent the sequence of locations using grid indexes. Given the limited number of possible index sequences, the rotation process ensures rotation invariance.

Through this process, our method achieves both translation and rotation invariance, ensuring that the model can robustly detect driving anomalies across diverse orientations and directions. Translation invariance is achieved by ensuring that the system produces the same response regardless of input shifts, while rotation invariance means that the model’s output remains consistent despite changes in the vehicle’s orientation. These properties make the proposed model highly adaptable and effective in detecting abnormal driving patterns under varying spatial conditions.

## III. PROPOSED APPROACH

In this section, we present our approach to detecting abnormal driving behaviors by integrating traditional naïve features with novel grid-indexed features. We utilize a multi-modal neural network model that combines both a Simple Neural Network (SNN) for Naive features and a Convolutional Neural Network (CNN) for grid-indexed features. This approach captures both temporal and spatial driving patterns, enhancing the accuracy and robustness of abnormal driving detection.

### A. MODEL

We developed a combined model leveraging naïve and grid-indexed telematics features. This methodology integrates simple and complex feature sets to improve the detection of abnormal driving behaviors.

#### 1) Naïve Data Model

The naïve dataset includes essential telematics metrics: distance (kilometers), speed (kph), speed over ground (SOG) (kph), and direction (azimuth). The azimuth value is mapped onto the unit circle and converted to corresponding coordinates to capture directional information effectively.

The naïve dataset features are processed through a Simple Neural Network (SNN) layout, as detailed in Table 2.

The input dimension for the naïve approach is 4, representing the four key telematics features: speed, SOG, direction, and distance. The model is trained using the Adam optimizer with a learning rate of 0.001 and CrossEntropyLoss as the loss function, suitable for binary classification tasks. Dropout layers with a rate of 0.5 are applied after the first and second fully connected layers to mitigate overfitting by randomly deactivating 50 percent of the neurons during training.

This configuration ensures that the model generalizes well to unseen data by not depending excessively on specific features.

#### 2) Grid-Based Data Model

The Grid-Based dataset organizes telematics data into a structured 2D grid to capture spatial patterns in driving behavior. Each grid cell represents a fixed area (e.g., 1 km by 1 km). As shown in Figure 1, we map the vehicle’s path onto a 3 × 3 grid. Each point along the vehicle’s path is assigned a grid cell based on its geographical coordinates (longitude and latitude). For instance, we start with an index of (0, 0) at the origin and update each cell to 1 if the vehicle passes through it, creating a binary matrix of movements. Larger grids provide more detailed tracking of spatial patterns.

The grid-based input is processed by a Convolutional Neural Network (CNN) to detect spatial dependencies. Table 3 provides an overview of the CNN layers, showing layer types, output shapes, activation functions, and dropout rates for each step.

- **Conv2D Layers:** Convolutional layers apply filters that help detect spatial features, such as changes in direction or dense areas of movement, within the grid.
- **MaxPooling2D Layers:** These layers downsample the spatial data from the Conv2D layers, reducing its size while retaining key features for further analysis.
- **Flatten Layer:** This layer converts the 2D spatial data into a 1D vector, which is then fed into the fully connected layers.
- **Fully Connected Layers:** These layers analyze the flattened data to identify complex spatial patterns. The final output is a 32-dimensional vector capturing essential driving features.
- **Dropout:** We apply a dropout rate of 0.5 to the first fully connected layer to prevent overfitting by randomly disabling 50 percent of the units during training.

#### 3) Proposed Combined Data Model

The Combined model’s architecture integrates outputs from the naïve and Grid-based streams, enhancing the model’s capability to discern complex patterns indicative of abnormal driving behavior. The architecture is detailed as follows:

##### a: Feature Integration:

- Intermediate Representations:
  - Naïve Data Stream: The Simple Neural Network (SNN) processes naïve features such as speed, direction, and distance, producing an intermediate feature vector.
  - Grid-based Data Stream: The Convolutional Neural Network (CNN) processes the grid-indexed features, producing an intermediate feature vector.
- Concatenation Layer: The intermediate representations from the Naïve and Grid-based streams concatenate to form a unified feature vector.

The Combined model integrates the outputs from both the naïve and Grid-based models by concatenating their intermediate representations into a 64-dimensional feature vector. This vector is passed through a series of fully connected layers to refine the feature space before making the final classification. Dropout with a rate of 0.5 is applied to the first fully connected layer to prevent overfitting by randomly setting 50 percent of the input units to zero during training.

The final output is a 2-dimensional vector representing the probability of each class (normal or abnormal driving behavior), computed using the softmax activation function.

This multi-modal approach effectively captures temporal patterns from the naïve features and spatial relationships from the Grid-Index features, enabling the model to make more accurate predictions.

## IV. EXPERIMENTAL EVALUATION

We conducted experiments to evaluate the performance of the proposed combined approach. The goal was to demonstrate the performance improvements by integrating naïve and grid-based features. We aimed to answer three key questions: (1) What is the effect of data size? (2) What is the effect of the neural network width(number of nodes)? (3) What is the effect of the number of the neural network model depth(hidden layers)?

### A. DATA COLLECTION

We collected a comprehensive dataset over 3.5 years from 200 drivers, including individuals with Mild Cognitive Impairment (MCI), to evaluate our approach. Participants were recruited through community outreach, targeting drivers aged 65 and older with valid driver’s licenses and insurance. Participants were initially screened using the Montreal Cognitive Assessment (MoCA) to assess their eligibility. Drivers with a MoCA score of 19 or higher were included in the study, ensuring cognitive baseline comparability.

**FIGURE 9:**
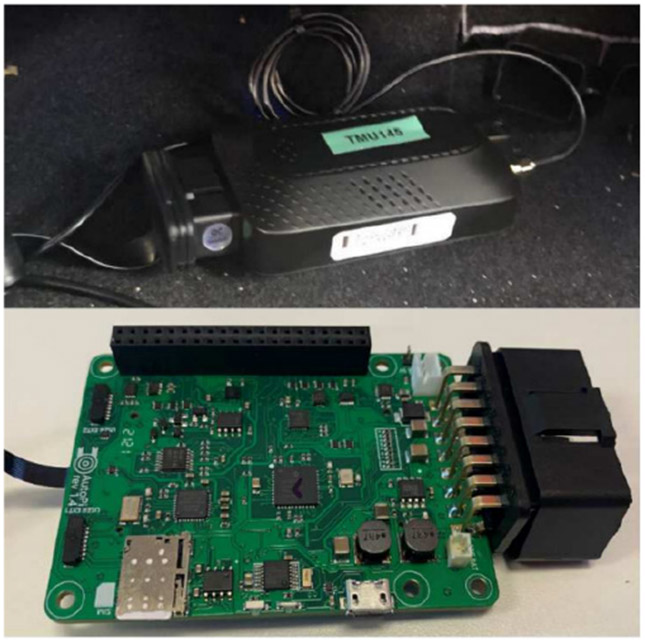
AutoPi device used for collecting telematics data, including speed, SOG, azimuth, distance, and GPS coordinates [26], [27].

We installed AutoPi devices in each participant’s vehicle to facilitate data collection. These devices continuously recorded critical telematics data throughout the study period, as illustrated in Figure 9. The AutoPi devices captured essential metrics such as vehicle speed (kph), speed over ground (SOG), direction (azimuth), distance, and GPS coordinates (longitude and latitude). This comprehensive data enabled a detailed analysis of driving behaviors related to cognitive impairment.

We labeled each trip segment based on the cognitive status of the drivers. For drivers diagnosed with MCI, we labeled all segments as abnormal (1), recognizing that abnormal drivers can exhibit both normal and abnormal driving behaviors. We expected the neural network to capture the frequency and pattern of these behaviors during training. This labeling approach enhanced the model’s ability to differentiate and accurately classify driving behaviors.

All participants provided informed consent, and the Institutional Review Board (IRB) approved the study. We conducted periodic assessments for the participants and compensated them for their participation. The dataset enabled us to train and evaluate our models on a diverse set of driving behaviors, supporting the effectiveness of our proposed approach.

### B. EXPERIMENT LAYOUT

The layout of our experiments is designed as follows:

#### a: Evaluation Metrics:

We used the following evaluation metrics to assess model performance:

- **Recall:** The proportion of actual positive instances that the model correctly identifies.
- **F1-Score:** Harmonic mean of precision and recall.
- **AUC:** Area Under the Receiver Operating Characteristic (ROC) curve.

#### b: Factors Analyzed:

Our experiments analyzed the impact of various factors, including:

- **Data Size:** Evaluating performance on datasets of varying sizes (4 months, 8 months, 1 year, and 2 years).
- **Number of Nodes:** Testing different configurations of node sizes in neural network layers.
- **Number of Hidden Layers:** Assessing the impact of network depth with different hidden layer configurations.

#### c: Approaches Compared:

We compared the following approaches in our experiments:

- **Naive Approach:** Using basic telematics features.
- **Grid-Index based Approach:** Incorporating spatial relationships through grid-indexed features.
- **Combined Approach:** Integrating both naïve and grid-based features.

### C. EXPERIMENT RESULTS

We experimentally evaluated the proposed algorithms by comparing the impact on the performance of (1) the data size, (2) the neural network width, and (3) the neural network height.

#### 1) Effect of Data Size

In the first experiment, we systematically varied the data sizes to evaluate their effect on the performance of the algorithms. We used performance metrics, including Recall, F1-Score, and AUC, to assess how well the models performed with different amounts of training data. We divided the dataset into subsets representing four months, eight months, one year, and two years of collected data. We then used each subset to train and test the model independently, allowing us to observe how increasing the amount of data affects model performance.

**FIGURE 11:**
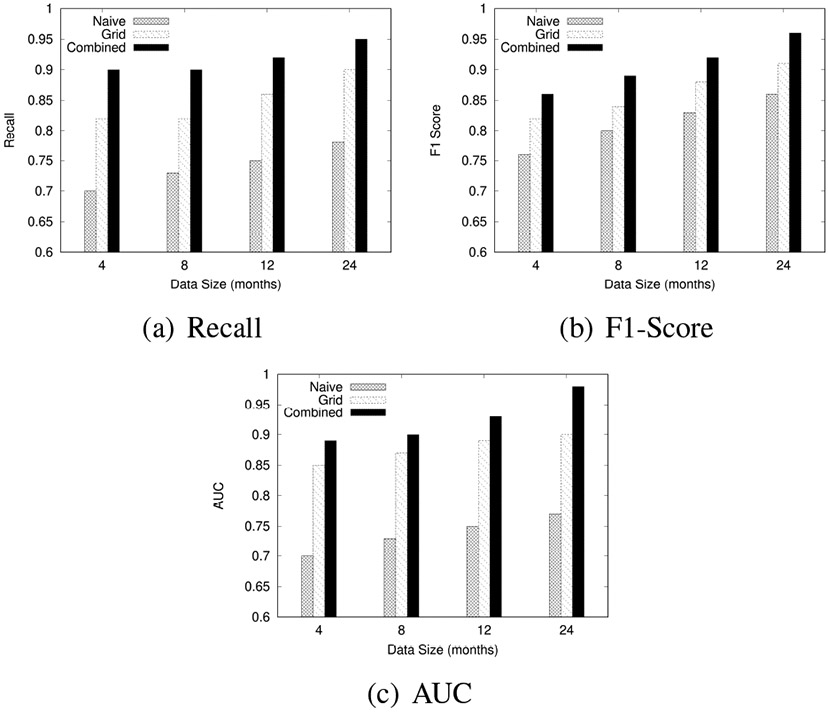
Effect of Data Size on Model Performance for F1-Score, AUC, and Recall across Naïve, Grid-based, and Combined approaches.

Figure 11 shows the results for Recall, F1-Scores, and AUC across the Naïve, Grid-based, and Combined approaches at these different data sizes.

As shown in Figure 11, increasing the dataset size leads to consistent improvements in model performance across all approaches. Larger datasets provide the models with more varied examples, which enhances their ability to generalize and accurately classify new, unseen data. This is reflected in the higher F1 scores, AUC values, and Recall observed as the data size increases. The model trained on the 2-year dataset achieves the highest performance, indicating the importance of a larger dataset in capturing complex driving behaviors.

#### 2) Effect of Neural Network Model Width (Number of Nodes)

In the second experiment, we evaluated how the number of nodes in the neural network layers affects model performance. We measured performance using Recall, F1-Score, and AUC. We conducted the experiments with different configurations of node sizes in the simple NN and CNN output layers while keeping other factors constant to isolate the effect of node variations.

**FIGURE 12:**
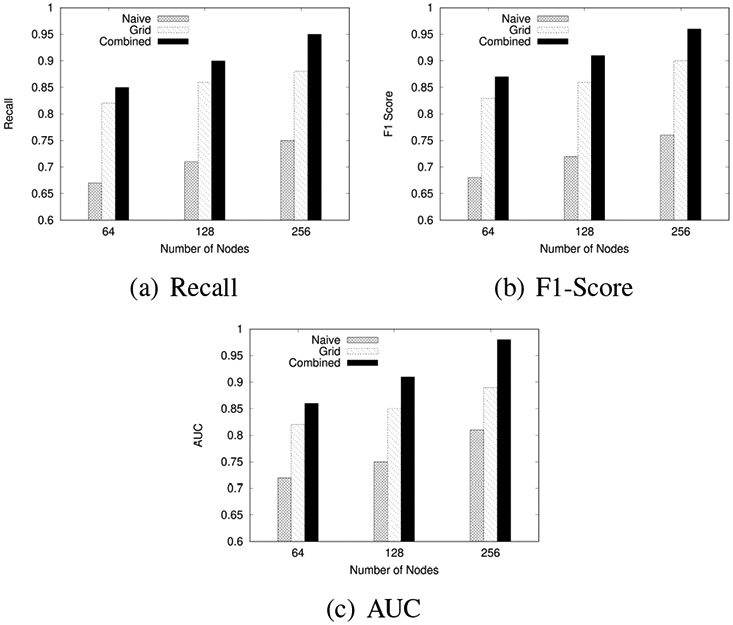
Effect of Number of Nodes on Model Performance for F1-Score, AUC, and Recall across Naïve, Grid-based, and Combined approaches.

Figure 12 shows the F1-Scores, AUC, and Recall for Naïve, Grid-based, and Combined approaches across varying node sizes:

The experiments reveal that increasing the number of nodes in the neural network layers generally enhances the model’s performance. Configurations with more nodes consistently achieve higher F1 scores, AUC values, and Recall. However, this trend may only sometimes be linear, as increasing the number of nodes beyond a certain point might lead to diminishing returns or even overfitting, depending on the dataset’s complexity and size.

#### 3) Effect of Neural Network Model Depth (Number of Layers)

The third experiment evaluated the effect of the number of hidden layers in the neural network on model performance. Performance measurements were Recall, F1-Score, and AUC. The experiments were conducted with 1, 2, and 3 hidden layer configurations while keeping the node size configuration fixed at 128 nodes for each layer to isolate the effect of varying the number of layers.

**FIGURE 13:**
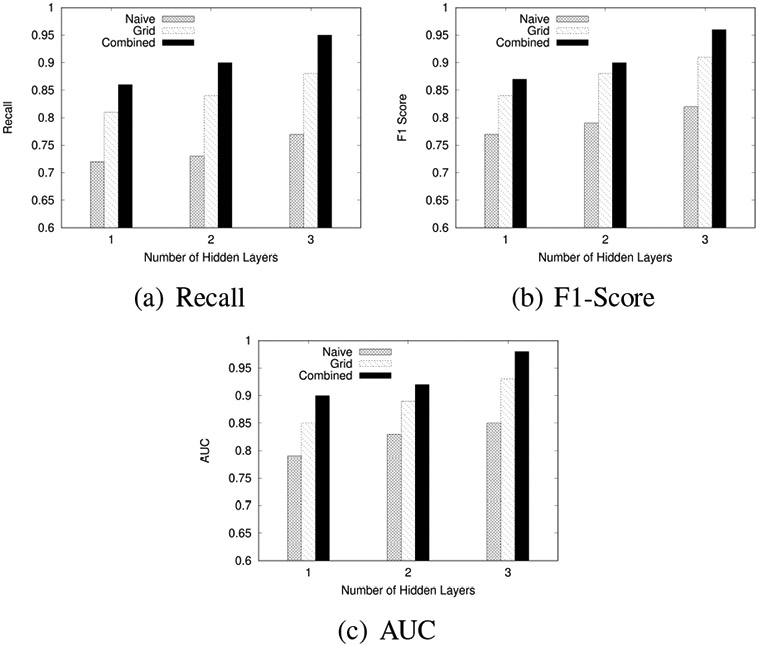
Effect of Layer Complexity on Model Performance for F1-Score, AUC, and Recall across Naïve, Grid-based, and Combined approaches.

Figure 13 shows the Recall, F1-Scores, and AUC for Naïve, Grid-based, and Combined approaches across varying numbers of hidden layers:

The results indicate that increasing the number of hidden layers improves model performance up to a certain point. The model with 3 hidden layers achieved the highest F1-Score, AUC, and Recall, indicating that deeper architectures can capture more complex data representations.

### D. EXPERIMENT ANALYSIS

After careful analysis, we determined that the best performance was achieved with the following configuration: 2 years of data, 128 nodes per layer, and 3 hidden layers. This configuration leverages the strengths identified in the previous experiments.

The Combined Approach achieves outstanding results, as shown in Table 6, with a Precision of 0.97, a Recall of 0.96, and an F1-Score of 0.96. These metrics highlight the approach’s robustness and accuracy in classifying driving behaviors.

The experimental results indicate that the Combined Approach significantly outperforms the Naive and Grid-based approaches across all evaluation metrics. The Naive Approach, while straightforward and easy to implement, frequently misclassifies data due to its reliance on basic telematics features, which fail to capture the complexity of driving behavior.

The Grid-based Approach offers a marked improvement by incorporating spatial relationships within the data, leading to better precision and recall. However, the Combined Approach truly excels by integrating the strengths of Naive and Grid-based features to deliver superior performance. To ensure the robustness and generalizability of the results, we employed 5-fold cross-validation during the evaluation process, which mitigates the risk of overfitting and validates the model’s performance across diverse subsets of the data. The higher precision, recall, and F1-Score demonstrate its ability to accurately identify abnormal driving behaviors while minimizing false positives and negatives.

In conclusion, the Combined Approach offers a robust and effective solution for detecting abnormal driving behaviors, leveraging the comprehensive insights gained from Naive and Grid-based features. This study’s findings pave the way for further research and development in telematics-based driving behavior analysis, with significant implications for road safety, fleet management, and insurance risk assessment.

### E. DISCUSSION

Our study highlights the significant improvements in detecting abnormal driving behavior by integrating diverse data features and advanced neural network architectures. The Naive Approach, while offering essential insights from telematics features, fails to capture the complex spatial and temporal dynamics of driving data, as shown by its lower precision and F1-Score.

The Grid-based Approach enhances detection by incorporating spatial relationships through grid-indexed features processed by Convolutional Neural Networks (CNNs). This method shows marked improvement over the Naive Approach, with higher precision and recall, indicating better identification of abnormal driving patterns. However, a comprehensive capture of driving behavior complexity is still needed.

The Combined Approach, integrating both Naive and Grid-based methods, achieves superior performance across all evaluation metrics. The high precision, recall, and F1-Score, along with a robust ROC curve, highlight its effectiveness in distinguishing normal and abnormal driving behaviors. This approach leverages the strengths of both Simple Neural Networks (SNN) and CNNs, resulting in a more accurate and generalizable model.

We experimented with additional features such as vehicle path straightness and speed fluctuations. However, these were ultimately excluded due to their limited effectiveness in capturing cognitive impairment indicators. Instead, we identified position, speed, direction, and distance traveled as the most relevant features for spatial-temporal analysis, as they best capture the driving patterns necessary for our study. Furthermore, our experiments confirmed that larger datasets, such as the two-year dataset, contribute significantly to model performance, enhancing the detection of subtle behavior changes.

These findings highlight the potential for implementing this classification model in real-world applications such as real-time monitoring systems. It can immediately detect anomalies, enhancing road safety for elderly drivers with Mild Cognitive Impairment (MCI) or dementia. Fleet management can also benefit from this model by optimizing driver behavior tracking and improving safety and operational efficiency. Additionally, insurance telematics can use the model’s anomaly detection capability to develop more tailored policies and perform better risk assessments. Our method is specifically designed to detect spatial-temporal patterns associated with cognitive impairment, focusing on behaviors such as erratic speed adjustments, lane deviations, and inconsistent following distances. This targeted detection enhances the model’s relevance for real-time monitoring systems that aim to improve road safety for drivers with MCI or dementia.

This study provides a foundation for more refined classification of driving behavior, paving the way for better insights into cognitive decline impacts on driving, and ultimately contributing to public safety, especially for vulnerable populations.

## V. CONCLUSION AND FUTURE WORK

Detecting abnormal driving behavior is essential for improving road safety, especially for drivers with Mild Cognitive Impairment (MCI) or dementia. This study presented a novel approach that combines traditional telematics features with grid-indexed spatial-temporal analysis, utilizing advanced neural network architectures.

The Naive Approach provided fundamental insights but was limited in capturing complex driving patterns, resulting in lower precision, recall, and F1-scores. The Grid-based Approach, by incorporating spatial relationships through Convolutional Neural Networks (CNNs), showed a marked improvement in precision and recall over the Naive Approach, yet still lacked a complete capture of driving behavior complexity.

The Combined Approach integrates naive and grid-based methods and shows superior performance across all evaluation metrics. It significantly increases precision, recall, and F1-Score compared to the baseline approaches, effectively distinguishing normal and abnormal driving behaviors. This approach leverages Simple Neural Networks (SNN) and CNNs to create a more accurate and generalizable model.

To promote reproducibility and facilitate further research, we have made the implementation code publicly available on GitHub at https://github.com/fiifijay/Spatial-Deep-Learning-to-Older-driver-Classification.

While our approach shows promise, computational demands, especially large datasets, remain challenging for real-time deployment. Future work could optimize efficiency through parallel processing techniques and explore lightweight model architectures for faster processing.

Future work may also consider integrating additional data sources, such as visual data from camera-based systems, to provide a more comprehensive view of driving behavior. Combining visual inputs with telematics and grid-based features could improve the model’s capability to detect subtle behavior variations, enhancing safety interventions for drivers with cognitive impairments.

This study provides a foundation for further exploration of spatial-temporal methods in driver behavior analysis, contributing valuable insights for applications in road safety, fleet management, and insurance risk assessment.

## Figures and Tables

**FIGURE 7:**
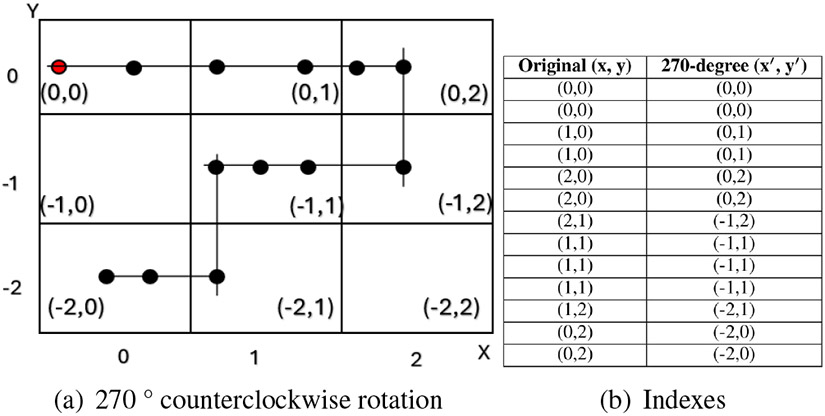
270 ° counterclockwise rotational transformation

**FIGURE 8:**
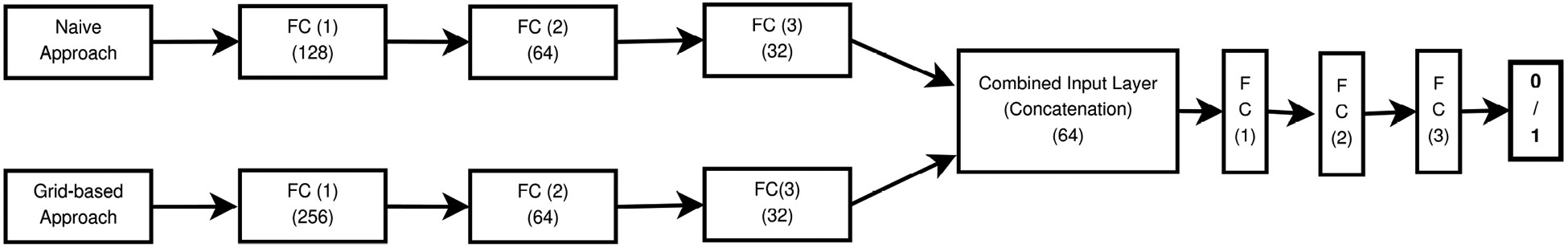
Architecture of the Combined Model integrating naïve and Grid-Indexed features. The model captures both temporal patterns from the naïve features and spatial relationships from the Grid-Index features.

**FIGURE 10:**
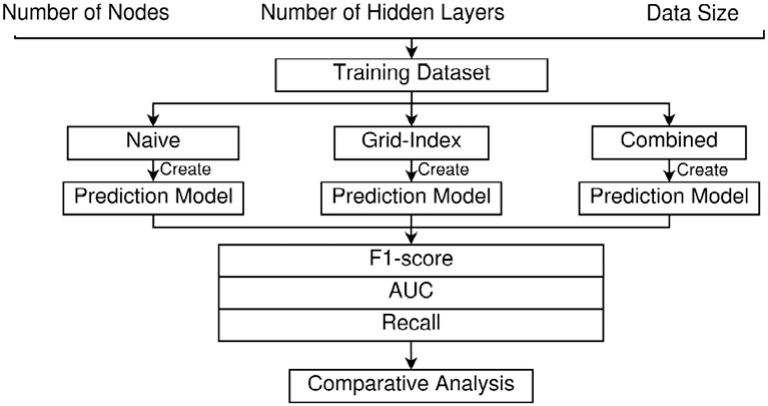
Experimental Layout

**TABLE 1:** Points and their Azimuth x and y values

Point	X	Y	Azimuth_x	Azimuth_y
1	0	0	0	1
2	0	0	0	1
3	1	0	0	1
4	1	0	0	1
5	2	0	1	0
6	2	0	1	0
7	2	1	0	−1
8	1	1	0	−1
9	1	1	1	0
10	1	1	1	0
11	1	2	0	−1
12	0	2	0	−1
13	0	2	0	0

**TABLE 2:** Naïve Data Stream Model

Layer (Type)	Output Shape	Activation	Dropout
Input Layer	(4)	-	-
Fully Connected Layer	(128)	ReLU	0.5
Fully Connected Layer	(64)	ReLU	0.5
Fully Connected Layer	(32)	ReLU	-
Output Layer	(2)	Softmax	-

**TABLE 3:** Layer Configuration Overview of the Grid-Based Data Stream

Layer(Type)	Shape	Activation	Dropout
Input Layer	(3, 3, 1)	-	-
Conv2D (32 filters, 3x3)	(1, 1, 32)	ReLU	-
MaxPooling2D (2x2)	(1, 1, 32)	-	-
Flatten	(32)	-	-
Fully Connected Layer	(256)	ReLU	0.5
Fully Connected Layer	(64)	ReLU	-
Fully Connected Layer	(32)	ReLU	-
Output	(32)	-	-

**TABLE 4:** Layer Configuration Overview of the Proposed Combined Model

Layer(Type)	Shape	Activation	Dropout
Concatenation Layer	(64)	-	-
Fully Connected Layer	(256)	ReLU	0.5
Fully Connected Layer	(64)	ReLU	-
Fully Connected Layer	(32)	ReLU	-
Output Layer	(2)	Softmax	-

**TABLE 5:** Experiment Configurations

Experiment	Data Size	Nodes Configuration	Hidden Layers
Exp 1	4 months	64, 32, 16	1
Exp 2	8 months	96, 48, 24	2
Exp 3	1 year	128, 64, 32	3
Exp 4	2 years	128, 64, 32	3

**TABLE 6:** Performance Metrics for Combined Approach

Metric	Precision	Recall	F1-Score
Combined Approach	0.97	0.96	0.96
